# Challenges and potential solutions of microalgae-based systems for wastewater treatment and resource recovery

**DOI:** 10.3389/fbioe.2023.1210228

**Published:** 2023-06-05

**Authors:** Linqing Li, Kun Gao, Mengting Yang, Qilin Zheng, Meng Zhang, Xiangyuan Deng

**Affiliations:** ^1^ College of Biotechnology, Jiangsu University of Science and Technology, Zhenjiang, China; ^2^ Key Laboratory of Ecological Impacts of Hydraulic-Projects and Restoration of Aquatic Ecosystem of Ministry of Water Resources, Institute of Hydroecology, Ministry of Water Resources and Chinese Academy of Sciences, Wuhan, China

**Keywords:** Microalage, wastewater treament, challenges, potential solutions, resource recovery

## 1 Introduction

Water is an essential resource for human survival and a pivotal feedstock for industrial production (Song et al., 2022). With the rapid development of industrialization, urbanization, and human population, excessive wastewater (about 380 billion t) would be generated annually across the world (Qadir et al., 2020). Wastewater contains various nutrient elements (e.g., nitrogen, phosphorus, organic compounds, and heavy metals), which can cause water eutrophication if it is released into natural water bodies without treatment (Wan Mahari et al., 2022). Under this situation, a wide range of wastewater treatment plants has been constructed for removing/reducing the nutrients to meet water quality regulations, but the traditional plants have some disadvantages, such as uneven removal efficiency, high installation cost, and diseconomy (Dutta et al., 2021). Especially, large amounts of greenhouse gases, mainly consisting of CO_2_, methane (CH_4_), and N_2_O, will be emitted into the atmosphere during the traditional wastewater treatment process. For example, emissions of greenhouse gases from municipal wastewater treatment plants in Beijing were estimated to be 1 045 611.5 t CO_2_-eq (tons of CO_2_-equivalent), with an average emission intensity of 0.603 kg CO_2_-eq/m^3^ (Zhou et al., 2022). Thus, greenhouse gases emitted from wastewater treatment plants have become a worldwide issue for the reduction of carbon footprint (Du et al., 2022).

**FIGURE 1:**
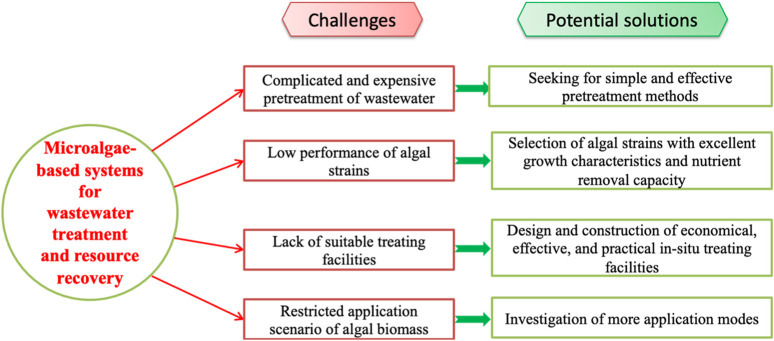
Challenges and potential solutions of microalgae-based systems for wastewater treatment and resource recovery.

At present, it has been widely recognized that microalgae are a promising renewable resource for wastewater treatment and resource recovery because they not only purify wastewater from different sources, but also use wastewater and CO_2_ as growth matrix to produce valuable biological products, biomaterials and bioenergy (Song et al., 2022; Srimongkol et al., 2022). Based on previous studies, some key opinions about this topic are summarized and listed as follows. Firstly, microalgae could remove/reduce nutrients, organic pollutants, heavy metals, chemical oxygen demand (COD), and biological oxygen demand (BOD) in wastewater effectively (Song et al., 2022). Secondly, algal biomass cultivated in wastewater could be used as a potential source of precious products that could be utilized in biofuels, cosmetics, pharmaceuticals, and nutraceuticals fields (Li et al., 2021; Lu and Lu, 2022; Lu and Xiao, 2022). Thirdly, the microalgae-driven wastewater treatment could help to realize the integration of wastewater treatment, industrial CO_2_ sequestration and microalgal biomass valorization, and shift wastewater treatment from linear economy to circular economy (Vaz et al., 2023). However, some limiting factors which may hinder the development of microalgae-driven wastewater treatment systems were neglected by previous studies. Herein, some challenges and potential solutions on the application of microalgae for wastewater treatment and resource recovery were proposed in this paper (Figure 1). It is hoped that the comments and discussions can prevent the overly optimistic attitudes towards the practical application of microalgae in wastewater treatment and resource recovery, and spur investigators to find out practically-feasible solutions to these challenging problems.

## 2 Wastewater treatment using microalgae: challenges and potential solutions

### 2.1 Complicated and expensive pretreatment of wastewater

Due to the different production materials and manufacturing technology, wastewater possesses different physicochemical properties. For example, most of nitrogen in silk industry wastewater is in the form of organic compounds, but that in swine manure is in the form of NH_4_^+^-N (Deng et al., 2017; Deng et al., 2020). Wastewater collected from different processing units of the silk industry (i.e., cooking cocoon wastewater, reeling wastewater, and frigon wastewater) have different physicochemical characteristics (Deng et al., 2020). Moreover, some toxic compounds, such as pesticides, insecticides, antibiotics, and toxic metals, are present in agricultural wastewater (Wang et al., 2018). Thus, most wastewater need to be pre-treated or diluted before its treatment using microalgae, which accounts a fraction of the treatment cost. The frequently-used pretreatment methods are anaerobic digestion, chemical oxidation, filtration, autoclave, and UV-radiation (Sharmila et al., 2020). In the future, it is urgent to seek for simple and effective pretreatment methods, which could be used to pretreat wastewater in low cost.

### 2.2 Low performance of algal strains

Different species of microalgae have different characteristics, such as nutrient removal efficiency, tolerance to toxic compounds, adaptability and cell composition (Lu et al., 2020). For example, López-Sánchez et al. (2022) summarized the maximum biomass concentration, COD removal, total nitrogen removal, and total phosphorus removal from different microalgae species cultivated in primary livestock wastewater as a medium, and pointed out that selection of an algal strain with high performance for wastewater treatment and resource recovery was very important. In future, selection of algal strains with excellent growth characteristics and nutrient removal capacity should be carried out by different methods, such as genetic engineering, adaptive laboratory evolution, and random mutagenesis (Hassanien et al., 2023).

### 2.3 Lack of suitable treating facilities

At present, the frequently-used microalgae cultivation facilities have been directly substituted into the field of microalgae-based wastewater treatment. For example, some open systems, such as waste stabilization ponds, raceway reactors, and sophisticated high-rate algal ponds, have been applied in this field because they are relatively inexpensive to build and easy to scale up (Leong et al., 2021). But the open systems have some main disadvantages, such as poor light utilization, evaporative losses, diffusion of CO_2_ to the atmosphere, contamination by predators, heterotrophs, and the requirement of large areas of land (Cheng et al., 2021). In order to address the above disadvantages, researchers have developed various closed photobioreactors for the treatment of wastewater using microalgae, such as vertical column, tubular, flat plate, membrane, and biofilm-based photobioreactors (Sathinathan et al., 2023). But their installation and maintenance costs are much higher than that of the open ones, suggesting that they are also not suitable facilities for wastewater management. Based on the above discussion, economical, effective, and practical *in situ* treating facilities have not been designed and constructed for microalgae-based wastewater treatment, indicating that more attentions should be paid to this field in future.

### 2.4 Restricted application scenario of algal biomass

It is well known that microalgae are rich in fatty acids, proteins, carbohydrates, lipids, and various micronutrients (e.g., vitamins, chlorophylls and carotenoids) (Tang et al., 2020). Nowadays, microalgae have been applied in various industrial areas, such as food, feed, pharmaceutical, and cosmetic industries, when they are cultivated in tap water, well water, and other clean water (Okeke et al., 2022). Studies on microalgae as animal feed and human food have been started since the early 1950s (Kusmayadi et al., 2021). In the past decades, microalgal biomass in the forms of capsule, powder, and tablet has merged in the market mainly as health food products (Kusmayadi et al., 2021). In 2018, the Food and Agriculture Organization of the United Nations (FAO) registered 87.0 tons of microalgae cultivation in 11 countries, with 86.6 tons only from China (Ferreira de Oliveira and Bragotto, 2022). However, the algal biomass could not be suitable to produce some typical products (e.g., food, feed, and biofertilizer) when microalgae grow on wastewater due to the exceeding permitted levels of heavy metals, pollutants, and pathogens. As reported previously, cadmium in the microalgal biomass was higher than the threshold established by the European regulation for fertilizing products, and three contaminants of emerging concern (hydrocinnamic acid, caffeine, and bisphenol A) were detected in the microalgae biomass used as biofertilizer (Álvarez-González et al., 2023). Although microalgae could remove pathogenic bacteria effectively from wastewater (Bhatt et al., 2023), *Escherichia coli* with a number of 400 CFU were found in 1 g algal biomass (Álvarez-González et al., 2023). In the light of above discussion, application scenario of algal biomass obtained from microalgae-based systems for wastewater treatment and resource recovery is restricted. Therefore, more and more application modes (e.g., algal biorefinery) should be investigated in the future.

## 3 Summary and recommendations

In recent years, the microalgae-based systems for wastewater treatment and resource recovery have attracted much attention because they could integrate wastewater treatment, industrial CO_2_ sequestration, and microalgal biomass valorization as a whole system. According to our research experiences and literature review, there are some problems challenging the application of microalgae in wastewater treatment and resource recovery. Firstly, pretreatment of wastewater is complicated when it is treated by using microalgae due to the fact that each type of wastewater has its own characteristics. Secondly, most of algal strains used to treat wastewater have low performances relative to traditional treatment methods. Thirdly, existing treating facilities cannot satisfy the demand for microalgae-based systems for wastewater treatment and resource recovery. Finally, application scenario of algal biomass obtained from the systems is restricted. Thus, there is still a long way to go for wastewater treatment using microalgae due to the above challenges, and the corresponding potential solutions have been recommended in this paper. It is expected that these recommendations could help investigators to find the future trends of this research topic.
